# Supplementary material for: The Evolution and Characterization of the RNA Interference Pathways in Lophotrochozoa
Source: Genome Biol Evol. 2024 May 7;16(5):evae098. doi: 10.1093/gbe/evae098 (PMC11114477; doi:10.1093/gbe/evae098)
Supplement: evae098_Supplementary_Data [file evae098_supplementary_data.zip › Supplementary_materials.zip!/selected_miRNAs/NoGe_selected_miRNAs/mirdeep_pdf_files/NMRB01000014.1_Notospermus_geniculatus_strain_Ushimado_scaffold14,_whole_genome_shotgun_sequence_619.pdf]

Provisional ID	:	NMRB01000014.1_Notospermus_geniculatus_strain_Ushimado_scaffold14,_whole_genome_shotgun_sequence_619
Score total	:	80990
Score for star read(s)	:	3.9
Score for read counts	:	80982.8
Score for mfe	:	0.3
Score for randfold	:	0
Score for cons. seed	:	3
Total read count	:	158856
Mature read count	:	158457
Loop read count	:	0
Star read count	:	399

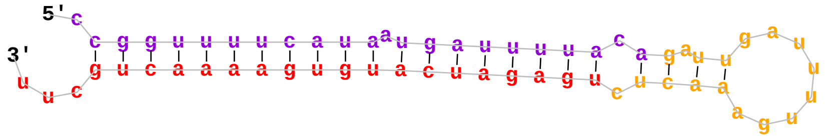

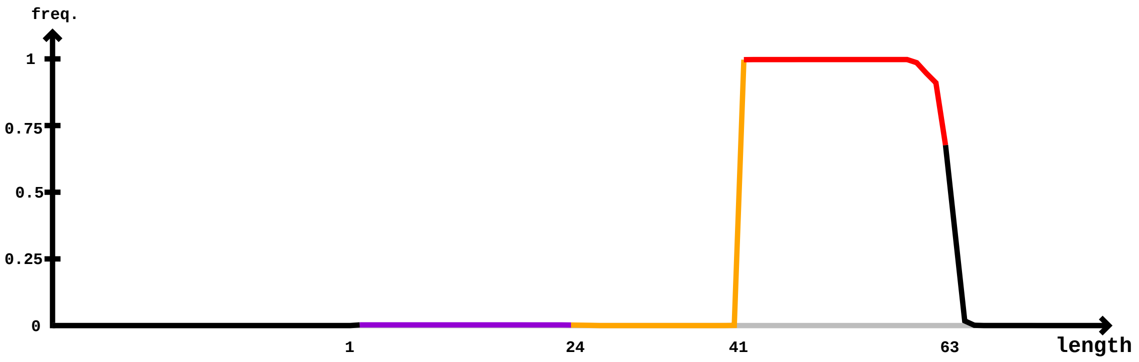

Star **Mature**

```
5'- ggacauggagcucuaccauaaaagacgaaaccgguuuucauaaugauuuuacagauugauuuugaaacucugagaucaugugaaaacugcuuuugucgacagugauca  -3'  obs
    ggacauggagcucuaccauaaaagacgaaaccgguuuucauaaugauuuuacagauugauuuugaaacucugagaucaugugaaaacugcuuuugucgacagugauca        exp
    ....((((.......))))....(((((((.(((((((((((.((((((((.((.((........)))).))))))))))))))))))).)))))))((......)).    reads   mm   sample
    ..............................ccgguuuucauaaugauA............................................................    1       1    Mm2
    ..............................ccgguuuucauaaugauuuu..........................................................    2       0    Mm2
    ..............................ccgguuuucauaaugauuuuac........................................................    5       0    Mm2
    ..............................ccgguuuucauaaugauuuuaca.......................................................    10      0    Mm2
    ..............................ccgguuuucauaaugauuuuacag......................................................    2       0    Mm2
    ..............................ccgguuuucauaaugauuuuacaga.....................................................    9       0    Mm2
    ..............................Ncgguuuucauaaugauuuuacaga.....................................................    1       1    Mm2
    ...................................................................cucugagaucaugugaaaacugcu.................    1       0    Mm2
    ....................................................................ucugagaucaugugaaaacugc..................    1       0    Mm2
    ....................................................................ucugagaucaugugaaaacugcu.................    1       0    Mm2
    .....................................................................cugagaucaugugaaaacugcu.................    11      0    Mm2
    .....................................................................cugagaucaugugaaUacugcuu................    1       1    Mm2
    .....................................................................cugagaucaugugaaaacugcuuu...............    1       0    Mm2
    .....................................................................cugagaucaugugaaaacugcuuuu..............    2       0    Mm2
    ......................................................................ugagaucaugugaaaacG....................    1       1    Mm2
    ......................................................................ugagaucaugugaaaaAu....................    1       1    Mm2
    ......................................................................ugagaucaugugaaaacu....................    106     0    Mm2
    ......................................................................ugagaucaugugaaaacGg...................    1       1    Mm2
    ......................................................................ugagaucaugugaaaaUug...................    1       1    Mm2
    ......................................................................ugagaucUugugaaaacug...................    2       1    Mm2
    ......................................................................ugagaucaugugaaaacCg...................    2       1    Mm2
    ......................................................................ugagaucaugugaaaacAg...................    1       1    Mm2
    ......................................................................ugagaucaugugaaaaAug...................    1       1    Mm2
    ......................................................................ugagaucaugugaaaacuA...................    15      1    Mm2
    ......................................................................ugagaucaugugaaaacug...................    213     0    Mm2
    ......................................................................ugagaucaugugaaaacuU...................    3       1    Mm2
    ......................................................................ugagaucaugCgaaaacugc..................    1       1    Mm2
    ......................................................................ugagaucaugugaaaacugU..................    3       1    Mm2
    ......................................................................ugagaucaugugaaaacugc..................    620     0    Mm2
    ......................................................................ugagaucaugugaaaacGgc..................    4       1    Mm2
    ......................................................................ugagaucaugAgaaaacugc..................    1       1    Mm2
    ......................................................................ugagaucaugugaaaaUugc..................    2       1    Mm2
    ......................................................................ugagaucaugugaaaacAgc..................    5       1    Mm2
```

Star Mature

ggacauggagcucuaccauaaaagacgaaaccgguuuucauaaugauuuuacagauugauuuugaaacucugagaucaugugaaaacugcuuuugucgacagugauca			
....ugUgaucaugugaaaacugc..................	1	1	Mm2
....ugagaucaugugaaaacugG..................	1	1	Mm2
....ugagGucaugugaaaacugc..................	1	1	Mm2
....ugagaucaugugaaaaAugc..................	3	1	Mm2
....ugagaucaugugaaaacCgc..................	4	1	Mm2
....ugagaucaugugaaaacugcC.................	3	1	Mm2
....ugagaucaugugaaaacugUu.................	2	1	Mm2
....ugagUucaugugaaaacugcu.................	1	1	Mm2
....ugagaucaugugaaaUcugcu.................	1	1	Mm2
....Cgagaucaugugaaaacugcu.................	2	1	Mm2
....ugagaucauAugaaaacugcu.................	1	1	Mm2
....ugagaucaugugaaaGcugcu.................	1	1	Mm2
....ugagaucaugugaaaacGgcu.................	29	1	Mm2
....uAagaucaugugaaaacugcu.................	2	1	Mm2
....ugagaucauCugaaaacugcu.................	1	1	Mm2
....ugagaucaugAgaaaacugcu.................	10	1	Mm2
....ugagaucauguUaaaacugcu.................	1	1	Mm2
....ugagaucaugugaaaacuUcu.................	1	1	Mm2
....ugagaucaugugaaaaUugcu.................	7	1	Mm2
....ugagaucaugugaaaaGugcu.................	6	1	Mm2
....ugagaucUugugaaaacugcu.................	3	1	Mm2
....ugagaucaCgugaaaacugcu.................	1	1	Mm2
....Agagaucaugugaaaacugcu.................	1	1	Mm2
....ugagaCcaugugaaaacugcu.................	1	1	Mm2
....ugagaucaugugaaGacugcu.................	1	1	Mm2
....ugagaucaugugaGaacugcu.................	3	1	Mm2
....ugagGucaugugaaaacugcu.................	1	1	Mm2
....ugagaucaugugaUaacugcu.................	1	1	Mm2
....ugagaucaugugaaaaAugcu.................	12	1	Mm2
....ugagaucaugGgaaaacugcu.................	4	1	Mm2
....ugagaucaugugUaaacugcu.................	1	1	Mm2
....ugCgaucaugugaaaacugcu.................	1	1	Mm2
....ugagaucaugCgaaaacugcu.................	5	1	Mm2
....ugagaucaugugaaaacugAu.................	1	1	Mm2
....ugagaAcaugugaaaacugcu.................	1	1	Mm2
....ugagaucaugugaaaacCgcu.................	22	1	Mm2
....ugGgaucaugugaaaacugcu.................	1	1	Mm2
....ugagaucaugugaaaacugcA.................	18	1	Mm2
....ugagaucaugugaaaacAgcu.................	25	1	Mm2
....ugagaucaugugaaaacugcu.................	4932	0	Mm2
....ugagaucaugugaaaacugcG.................	12	1	Mm2
....Ngagaucaugugaaaacugcu.................	3	1	Mm2
....ugagaucaugugaaaacuCcuu................	1	1	Mm2
....ugagaucaugugaaaacugcuA................	22	1	Mm2
....ugagaucauguUaaaacugcuu................	2	1	Mm2
....uUagaucaugugaaaacugcuu................	3	1	Mm2
....ugagaucaugugaaaacuUcuu................	8	1	Mm2
....ugagaucaugugaaaGcugcuu................	1	1	Mm2
....ugagaucaugugaaaaAugcuu................	77	1	Mm2
....ugagaucaugCgaaaacugcuu................	17	1	Mm2
....Cgagaucaugugaaaacugcuu................	4	1	Mm2
....ugagaucaugugaGaacugcuu................	4	1	Mm2
....Agagaucaugugaaaacugcuu................	7	1	Mm2
....ugagaucaugugaaaacugcCu................	8	1	Mm2
....ugagaucaugugGaaacugcuu................	3	1	Mm2
....uCagaucaugugaaaacugcuu................	1	1	Mm2
....ugCgaucaugugaaaacugcuu................	2	1	Mm2
....ugagaucaugugaaaacugcuu................	18640	0	Mm2
....ugagaucGugugaaaacugcuu................	3	1	Mm2
....ugagaucaCgugaaaacugcuu................	3	1	Mm2
....ugagaucaugAgaaaacugcuu................	33	1	Mm2
....ugagaucaugugaaaacAgcuu................	92	1	Mm2
....ugagaucauguAaaaacugcuu................	1	1	Mm2
....ugGgaucaugugaaaacugcuu................	4	1	Mm2
....ugagaucaugugaaaacGgcuu................	93	1	Mm2
....ugagauAaugugaaaacugcuu................	1	1	Mm2
....ugagaucaugugUaaacugcuu................	1	1	Mm2
....ugagaucaugugaaaacCgcuu................	112	1	Mm2
....ugagaucaugugaaCacugcuu................	1	1	Mm2
....ugagaucauAugaaaacugcuu................	3	1	Mm2

Star Mature

```
ggacauggagcucuaccauaaaagacgaaaccgguuuucauaaugauuuuacagauugauuuugaaacucugagaucaugugaaaacugcuuuugucgacagugauca
......................................................................Ngagaucaugugaaaacugcuu................   7     1   Mm2
......................................................................ugagaucauguCaaaacugcuu................   2     1   Mm2
......................................................................ugagCucaugugaaaacugcuu................   1     1   Mm2
......................................................................ugagaAcaugugaaaacugcuu................   3     1   Mm2
......................................................................Ggagaucaugugaaaacugcuu................   1     1   Mm2
......................................................................ugagaucaugugaCaacugcuu................   1     1   Mm2
......................................................................ugagaucaugugaUaacugcuu................   2     1   Mm2
......................................................................ugaAaucaugugaaaacugcuu................   2     1   Mm2
......................................................................ugagaucaugugaaaacugcGu................   2     1   Mm2
......................................................................uAagaucaugugaaaacugcuu................   6     1   Mm2
......................................................................ugagaucUugugaaaacugcuu................   2     1   Mm2
......................................................................ugagaucaugugaaaaGugcuu................   19    1   Mm2
......................................................................ugagaCcaugugaaaacugcuu................   3     1   Mm2
......................................................................ugagaNcaugugaaaacugcuu................   2     1   Mm2
......................................................................ugagaucaugGgaaaacugcuu................   23    1   Mm2
......................................................................ugagaucaugugaaaUcugcuu................   4     1   Mm2
......................................................................ugagaucaugugaaaacugcuG................   11    1   Mm2
......................................................................ugUgaucaugugaaaacugcuu................   3     1   Mm2
......................................................................ugagaucaugugaaGacugcuu................   1     1   Mm2
......................................................................ugagaucaugugaaUacugcuu................   1     1   Mm2
......................................................................ugagaucaugugaaaaUugcuu................   40    1   Mm2
......................................................................ugagaucaugugaaaacuAcuu................   1     1   Mm2
......................................................................ugagUucaugugaaaacugcuu................   1     1   Mm2
......................................................................ugagaucaugugaaaacugUuu................   9     1   Mm2
......................................................................ugagauUaugugaaaacugcuu................   6     1   Mm2
......................................................................ugagGucaugugaaaacugcuu................   2     1   Mm2
......................................................................ugagaucaugugaaaacugcuC................   6     1   Mm2
......................................................................ugagaucaAgugaaaacugcuu................   1     1   Mm2
......................................................................ugagaucaugugaaaacugAuu................   2     1   Mm2
......................................................................ugagaucaugugaaaacugcuuu...............   2484  0   Mm2
......................................................................ugagaucauAugaaaacugcuuu...............   1     1   Mm2
......................................................................Cgagaucaugugaaaacugcuuu...............   1     1   Mm2
......................................................................Ngagaucaugugaaaacugcuuu...............   3     1   Mm2
......................................................................ugGgaucaugugaaaacugcuuu...............   1     1   Mm2
......................................................................ugagaucaugugaaGacugcuuu...............   1     1   Mm2
......................................................................ugCgaucaugugaaaacugcuuu...............   1     1   Mm2
......................................................................Ggagaucaugugaaaacugcuuu...............   1     1   Mm2
......................................................................ugagaucUugugaaaacugcuuu...............   1     1   Mm2
......................................................................ugagaucaugugaaaacGgcuuu...............   11    1   Mm2
......................................................................ugagauUaugugaaaacugcuuu...............   1     1   Mm2
......................................................................ugagaucaugugaaaacCgcuuu...............   9     1   Mm2
......................................................................ugagaucaugugaaaaAugcuuu...............   18    1   Mm2
......................................................................ugagaucaugAgaaaacugcuuu...............   4     1   Mm2
......................................................................ugagaucaugugaaaacugcuuA...............   159   1   Mm2
......................................................................ugagaucaugugaaaacugcCuu...............   4     1   Mm2
......................................................................ugagaucaugugaaaacAgcuuu...............   10    1   Mm2
......................................................................ugagaucaGgugaaaacugcuuu...............   1     1   Mm2
......................................................................ugagaucaugCgaaaacugcuuu...............   3     1   Mm2
......................................................................ugagaucaugugaaaacugcuuC...............   9     1   Mm2
......................................................................ugagaucaugugaaUacugcuuu...............   2     1   Mm2
......................................................................ugagaucaugugaaaaGugcuuu...............   6     1   Mm2
......................................................................uCagaucaugugaaaacugcuuu...............   2     1   Mm2
......................................................................ugagaucaugugaaaacugUuuu...............   2     1   Mm2
......................................................................ugagaucaugugaaaaUugcuuu...............   6     1   Mm2
......................................................................ugagaucaugugaaaacugcuuG...............   1     1   Mm2
......................................................................ugagaucaCgugaaaacugcuuu...............   1     1   Mm2
......................................................................ugaAaucaugugaaaacugcuuu...............   1     1   Mm2
......................................................................ugagaucaugugaaaacugcuGu...............   1     1   Mm2
......................................................................ugagaucaugGgaaaacugcuuu...............   2     1   Mm2
......................................................................ugagauGaugugaaaacugcuuu...............   1     1   Mm2
......................................................................ugagaucaugugaaaaUugcuuuu..............   4     1   Mm2
......................................................................ugagaucaugAgaaaacugcuuuu..............   1     1   Mm2
......................................................................ugagaucaugugaaaacugcuuAu..............   19    1   Mm2
......................................................................ugagaucaugugaaaacGgcuuuu..............   2     1   Mm2
......................................................................ugagaucaugugaaaacCgcuuuu..............   1     1   Mm2
......................................................................uAagaucaugugaaaacugcuuuu..............   1     1   Mm2
......................................................................ugagaucaugugaaaacugcuuuG..............   1     1   Mm2
......................................................................ugagaucaugugaaaacugUuuuu..............   2     1   Mm2
......................................................................ugagaucaugugaaaacAgcuuuu..............   2     1   Mm2
......................................................................ugagaucaugugaaaacugcuuuA..............   11    1   Mm2
```

```
                              Star                                    Mature
ggacauggagcucuaccauaaaagacgaaaccgguuuucauaaugauuuuacagauugauuuugaaacucugagaucaugugaaaacugcuuuugucgacagugauca
......................................................................ugagaucaugugaaaacugcuuGu..............   1      1     Mm2
......................................................................ugagaucaugugaaaaAugcuuu...............   3      1     Mm2
......................................................................Ngagaucaugugaaaacugcuuu...............   1      1     Mm2
......................................................................ugagaucaugugaaaacugcuuCu..............   2      1     Mm2
......................................................................ugagaucaugugaaaacugcuuu...............   451    0     Mm2
......................................................................ugagaucaugugaaaacugcuuuU..............   26     1     Mm2
.......................................................................gagaucaugugaaaacugcu.................   1      0     Mm2
.......................................................................gagaucaugugaaaacugcuu................   2      0     Mm2
.......................................................................gagaucauguCaaaacugcuu................   1      1     Mm2
.......................................................................gagaucaugugaaaacugcuuu...............   2      0     Mm2
........................................................................agaucaugugaaaacugcuu................   1      0     Mm2
........................................................................agaucaugugaaaacugcuuu...............   1      0     Mm2
.........................................................................gaucaugugaaaacugcuu................   1      0     Mm2

..............................ccgguuuucauaaugauuuuac........................................................   1      0     Mm1
......................................................................ugagaucaugugaaaacA....................   2      1     Mm1
......................................................................ugagaucaugugaaaacu....................   63     0     Mm1
......................................................................ugagaucaugugaaaacC....................   1      1     Mm1
......................................................................ugagaAcaugugaaaacu....................   1      1     Mm1
......................................................................ugagaucaugAgaaaacug...................   1      1     Mm1
......................................................................ugagaucaugugaaaacGg...................   3      1     Mm1
......................................................................ugagaucaugugaaaaUug...................   3      1     Mm1
......................................................................ugagaucaugugaGaacug...................   1      1     Mm1
......................................................................ugagaucaugGgaaaacug...................   1      1     Mm1
......................................................................ugagaucaugugaaaacuA...................   1      1     Mm1
......................................................................ugagaucaugugaaaacuU...................   12     1     Mm1
......................................................................ugagaucaugugaaaacug...................   191    0     Mm1
......................................................................ugagaucaugugaaaacCg...................   2      1     Mm1
......................................................................ugagaucaugugaaaacugU..................   16     1     Mm1
......................................................................ugagaucaugugaaaacugc..................   250    0     Mm1
......................................................................ugagauUaugugaaaacugc..................   1      1     Mm1
......................................................................ugagaucaugugaaaaAugc..................   2      1     Mm1
......................................................................ugagaucaugugaaaacGgc..................   2      1     Mm1
......................................................................ugagaucaugugaaaacugcu.................   1465   0     Mm1
......................................................................ugagaucaugugaaaGcugcu.................   1      1     Mm1
......................................................................ugagaNcaugugaaaacugcu.................   1      1     Mm1
......................................................................ugagaucaugGgaaaacugcu.................   1      1     Mm1
......................................................................ugagaucaugugaaaacugUu.................   1      1     Mm1
......................................................................ugagaucaugugaaaacGgcu.................   9      1     Mm1
......................................................................uUagaucaugugaaaacugcu.................   1      1     Mm1
......................................................................ugagaucaugugaaaaUugcu.................   2      1     Mm1
......................................................................ugagGucaugugaaaacugcu.................   1      1     Mm1
......................................................................ugagaucaugugaaaaAugcu.................   7      1     Mm1
......................................................................ugagauGaugugaaaacugcu.................   2      1     Mm1
......................................................................ugagaucauguCaaaacugcu.................   1      1     Mm1
......................................................................ugagaucaugAgaaaacugcu.................   1      1     Mm1
......................................................................ugagaucaugugaaaacCgcu.................   5      1     Mm1
......................................................................ugagCucaugugaaaacugcu.................   1      1     Mm1
......................................................................ugagaucaugugaaaCcugcu.................   2      1     Mm1
......................................................................ugagaucaCgugaaaacugcu.................   1      1     Mm1
......................................................................ugagaucaugugaaaacAgcu.................   8      1     Mm1
......................................................................ugagaucaugugaaaaGugcu.................   1      1     Mm1
......................................................................ugagaucaAgugaaaacugcu.................   1      1     Mm1
......................................................................ugagaucauguCaaaacugcuu................   1      1     Mm1
......................................................................ugagaucaugugaaaacGgcuu................   16     1     Mm1
......................................................................uAagaucaugugaaaacugcuu................   1      1     Mm1
......................................................................ugagaucaCgugaaaacugcuu................   2      1     Mm1
......................................................................ugagaucaugugaCaacugcuu................   1      1     Mm1
......................................................................ugagaucaugugaaaacAgcuu................   25     1     Mm1
......................................................................ugaCaucaugugaaaacugcuu................   1      1     Mm1
......................................................................ugagaucaugugaaaaAugcuu................   10     1     Mm1
......................................................................ugagauUaugugaaaacugcuu................   1      1     Mm1
......................................................................ugagaucGugugaaaacugcuu................   1      1     Mm1
......................................................................ugagaucaugugaaaacugUuu................   13     1     Mm1
......................................................................ugagaucaugugaaaacCgcuu................   14     1     Mm1
......................................................................uCagaucaugugaaaacugcuu................   1      1     Mm1
......................................................................ugagaCcaugugaaaacugcuu................   1      1     Mm1
......................................................................Ngagaucaugugaaaacugcuu................   1      1     Mm1
......................................................................ugagaucaugugaaaacugcuu................   3492   0     Mm1
......................................................................ugCgaucaugugaaaacugcuu................   1      1     Mm1
```

```
                              Star                                      Mature
ggacauggagcucuaccauaaaagacgaaaccgguuuucauaaugauuuuacagauugauuuugaaacucugagaucaugugaaaacugcuuuugucgacagugauca
......................................................................ugUgaucaugugaaaacugcuu................    3    1    Mm1
......................................................................ugaAaucaugugaaaacugcuu................    1    1    Mm1
......................................................................ugagaucaugugaaaacuAcuu................    2    1    Mm1
......................................................................ugagaucaugugaaaaUugcuu................    10   1    Mm1
......................................................................ugagaucauAugaaaacugcuu................    1    1    Mm1
......................................................................ugagaucaugGgaaaacugcuu................    5    1    Mm1
......................................................................ugagUucaugugaaaacugcuu................    1    1    Mm1
......................................................................ugagaucUugugaaaacugcuu................    1    1    Mm1
......................................................................ugagaucaugAgaaaacugcuu................    11   1    Mm1
......................................................................ugagaucaugugaGaacugcuu................    2    1    Mm1
......................................................................ugagaucaugugGaaacugcuu................    1    1    Mm1
......................................................................ugagaucaugugaaaaGugcuu................    3    1    Mm1
......................................................................ugagaucaugugaaaacuUcuu................    1    1    Mm1
......................................................................ugagaucauguAaaaacugcuu................    1    1    Mm1
......................................................................ugagaucaugCgaaaacugcuu................    2    1    Mm1
......................................................................ugagaucaugugaaaacGgcuuu...............    2    1    Mm1
......................................................................ugagaucaugugaaaacCgcuuu...............    2    1    Mm1
......................................................................ugagaucaugugaaaaAugcuuu...............    3    1    Mm1
......................................................................ugagaucaugCgaaaacugcuuu...............    1    1    Mm1
......................................................................ugagaucaugAgaaaacugcuuu...............    1    1    Mm1
......................................................................ugagaucaugugaaaacugcuuA...............    23   1    Mm1
......................................................................ugagaNcaugugaaaacugcuuu...............    1    1    Mm1
......................................................................ugagaucaugugaaaacugcuuu...............    438  0    Mm1
......................................................................ugagaucaugugGaaacugcuuu...............    1    1    Mm1
......................................................................ugagaucaugugaaaacugUuuu...............    4    1    Mm1
......................................................................ugagaucaugugaaaacAgcuuu...............    1    1    Mm1
......................................................................Ngagaucaugugaaaacugcuuu...............    1    1    Mm1
......................................................................ugagaucaugugaaaaUugcuuu...............    4    1    Mm1
......................................................................ugagaucaugugaaaacugcuuuu..............    70   0    Mm1
......................................................................ugagaucaugugaaaacugcuAuu..............    4    1    Mm1
......................................................................ugGgaucaugugaaaacugcuuuu..............    1    1    Mm1
......................................................................ugagaucaugugaaaacugcuuGu..............    4    1    Mm1
......................................................................ugagaucaugugaaaacugcuuuuU.............    1    1    Mm1
......................................................................ugagaucaugugaaaacugcuuuuUu............    1    1    Mm1

..............................ccgguuuucauaaugauu............................................................    1    0    Mm3
..............................ccgguuuucauaaugauuuuac........................................................    9    0    Mm3
..............................ccgguuuucauaaugauuuuaca.......................................................    6    0    Mm3
..............................ccgguuuucauaaugauuuuacag......................................................    2    0    Mm3
..............................ccgguuuucauaaugauuuuacaga.....................................................    3    0    Mm3
..............................ccgguuuucauaaugauuuuacagauug..................................................    1    0    Mm3
.....................................................................cugagaucaugugaaaacugcu.................    6    0    Mm3
.....................................................................cugagaucaugugaaaacugcuu................    5    0    Mm3
.....................................................................cugagaucaugugaaaacugcuuu...............    2    0    Mm3
......................................................................ugagaucaugugaaaacu....................    96   0    Mm3
......................................................................ugagaucaugugaaaacG....................    1    1    Mm3
......................................................................ugagaucaugugaaaacC....................    1    1    Mm3
......................................................................ugagaucaugugaaaacCg...................    1    1    Mm3
......................................................................ugagaucaugugaaaacuA...................    14   1    Mm3
......................................................................ugagaucaugugaaaacuU...................    5    1    Mm3
......................................................................ugagaucaugugaaaaAug...................    1    1    Mm3
......................................................................ugagaucaugugaaaacug...................    122  0    Mm3
......................................................................ugagaucaugugaaaacAgc..................    1    1    Mm3
......................................................................ugagaucaugugaaaacugc..................    579  0    Mm3
......................................................................ugagaucaugugGaaacugc..................    1    1    Mm3
......................................................................ugagaucaugugaaaacGgc..................    2    1    Mm3
......................................................................ugagaucaugugaaaaAugc..................    6    1    Mm3
......................................................................ugagaucaugugaaaacugU..................    8    1    Mm3
......................................................................ugagauAaugugaaaacugc..................    1    1    Mm3
......................................................................ugagaucaugCgaaaacugc..................    1    1    Mm3
......................................................................ugagaucaugugaaaacCgc..................    3    1    Mm3
......................................................................Ngagaucaugugaaaacugc..................    1    1    Mm3
......................................................................ugagaucauAugaaaacugcu.................    1    1    Mm3
......................................................................ugGgaucaugugaaaacugcu.................    1    1    Mm3
......................................................................ugagauUaugugaaaacugcu.................    2    1    Mm3
......................................................................ugagaucaugGgaaaacugcu.................    3    1    Mm3
......................................................................ugagaucaugugaGaacugcu.................    1    1    Mm3
......................................................................Agagaucaugugaaaacugcu.................    1    1    Mm3
......................................................................ugagaNcaugugaaaacugcu.................    1    1    Mm3
......................................................................ugagaucaugugaaaUcugcu.................    1    1    Mm3
```

Star **Mature**

```
ggacauggagcucuaccauaaaagacgaaaccgguuuucauaaugauuuuacagauugauuuugaaacucugagaucaugugaaaacugcuuuugucgacagugauca
......................................................................ugagaucaugugaaaacCgcu.................	27	1	Mm3
......................................................................ugagCucaugugaaaacugcu.................	2	1	Mm3
......................................................................ugagaucauguUaaaacugcu.................	1	1	Mm3
......................................................................Cgagaucaugugaaaacugcu.................	1	1	Mm3
......................................................................ugagaucaugugaaaGcugcu.................	1	1	Mm3
......................................................................ugagaucaugugaaaacugcG.................	11	1	Mm3
......................................................................ugagaucaugugaaaaAugcu.................	22	1	Mm3
......................................................................ugagaucaugAgaaaacugcu.................	12	1	Mm3
......................................................................ugagaucaugugaaaacAgcu.................	29	1	Mm3
......................................................................ugagaucaugugaaaacugcC.................	5	1	Mm3
......................................................................Ngagaucaugugaaaacugcu.................	2	1	Mm3
......................................................................ugagaucaugugaaaaUugcu.................	13	1	Mm3
......................................................................ugaAaucaugugaaaacugcu.................	1	1	Mm3
......................................................................ugagaucaugugaaaaGugcu.................	5	1	Mm3
......................................................................ugagaucaugugaUaacugcu.................	1	1	Mm3
......................................................................ugagaucaugugaaaacGgcu.................	24	1	Mm3
......................................................................ugagaucaugugaaUacugcu.................	3	1	Mm3
......................................................................ugagaucaugugaaaacugUu.................	3	1	Mm3
......................................................................ugagaucaugugaaaacugcA.................	23	1	Mm3
......................................................................ugagaucGugugaaaacugcu.................	3	1	Mm3
......................................................................ugagaucaugugaaaacugcu.................	5317	0	Mm3
......................................................................uAagaucaugugaaaacugcu.................	2	1	Mm3
......................................................................ugagaucaugCgaaaacugcu.................	10	1	Mm3
......................................................................ugagaucaugugaaGacugcu.................	2	1	Mm3
......................................................................ugagaucaCgugaaaacugcu.................	1	1	Mm3
......................................................................ugagaucaugugaCaacugcu.................	1	1	Mm3
......................................................................ugagauUaugugaaaacugcuu................	5	1	Mm3
......................................................................ugagaucaugugaaaUcugcuu................	1	1	Mm3
......................................................................ugagaucaugugaaaacAgcuu................	82	1	Mm3
......................................................................ugCgaucaugugaaaacugcuu................	1	1	Mm3
......................................................................ugagaucaugugaaaacuUcuu................	2	1	Mm3
......................................................................ugagaucaugugaaaacugUuu................	10	1	Mm3
......................................................................ugagaucaugugaaaaAugcuu................	58	1	Mm3
......................................................................ugagauNaugugaaaacugcuu................	2	1	Mm3
......................................................................ugagaucaugugaaaacuAcuu................	1	1	Mm3
......................................................................ugagaucauguUaaaacugcuu................	2	1	Mm3
......................................................................uAagaucaugugaaaacugcuu................	5	1	Mm3
......................................................................uCagaucaugugaaaacugcuu................	1	1	Mm3
......................................................................ugagaucaugugUaaacugcuu................	2	1	Mm3
......................................................................ugagaucaugugaaGacugcuu................	1	1	Mm3
......................................................................ugagaucaugugaaaaUugcuu................	24	1	Mm3
......................................................................ugagaucaugugaUaacugcuu................	1	1	Mm3
......................................................................ugagaucaugugaGaacugcuu................	4	1	Mm3
......................................................................ugagaucaugugaaaacugcuu................	13768	0	Mm3
......................................................................Cgagaucaugugaaaacugcuu................	1	1	Mm3
......................................................................Ngagaucaugugaaaacugcuu................	8	1	Mm3
......................................................................ugagaucaugAgaaaacugcuu................	33	1	Mm3
......................................................................ugagaucaugugaaaacGgcuu................	60	1	Mm3
......................................................................ugGgaucaugugaaaacugcuu................	10	1	Mm3
......................................................................ugagaucaugugGaaacugcuu................	1	1	Mm3
......................................................................ugagaucauCugaaaacugcuu................	2	1	Mm3
......................................................................ugagaucaugugCaaacugcuu................	1	1	Mm3
......................................................................ugagGucaugugaaaacugcuu................	3	1	Mm3
......................................................................ugaUaucaugugaaaacugcuu................	1	1	Mm3
......................................................................ugagaucaugugaaaacugcCu................	10	1	Mm3
......................................................................ugagaucauguAaaaacugcuu................	2	1	Mm3
......................................................................Agagaucaugugaaaacugcuu................	2	1	Mm3
......................................................................ugagaucaugugaaaacugGuu................	1	1	Mm3
......................................................................ugagaucaugugaaaacugcuA................	27	1	Mm3
......................................................................ugagCucaugugaaaacugcuu................	6	1	Mm3
......................................................................ugagaAcaugugaaaacugcuu................	2	1	Mm3
......................................................................ugagaucUugugaaaacugcuu................	3	1	Mm3
......................................................................ugagaucauUugaaaacugcuu................	3	1	Mm3
......................................................................ugagaucaugugaaaacugcuC................	1	1	Mm3
......................................................................ugagaucaugugaCaacugcuu................	1	1	Mm3
......................................................................uUagaucaugugaaaacugcuu................	1	1	Mm3
......................................................................Ggagaucaugugaaaacugcuu................	1	1	Mm3
......................................................................ugagaucaAgugaaaacugcuu................	1	1	Mm3
......................................................................ugNgaucaugugaaaacugcuu................	1	1	Mm3
......................................................................ugagaucauguCaaaacugcuu................	1	1	Mm3
```

```
                              Star                                    Mature
ggacauggagcucuaccauaaaagacgaaaccgguuuucauaaugauuuuacagauugauuuugaaacucugagaucaugugaaaacugcuuuugucgacagugauca
......................................................................ugagaucaugCgaaaacugcuu................ 10 1 Mm3
......................................................................ugagUucaugugaaaacugcuu................ 2 1 Mm3
......................................................................ugagaucaugugaaaacCgcuu................ 81 1 Mm3
......................................................................ugagaucaugugaaaaGugcuu................ 14 1 Mm3
......................................................................ugagaucaugugaaaacugcuG................ 5 1 Mm3
......................................................................ugagaucGugugaaaacugcuu................ 4 1 Mm3
......................................................................ugagaucaugugaaaGcugcuu................ 1 1 Mm3
......................................................................ugaAaucaugugaaaacugcuu................ 1 1 Mm3
......................................................................ugagaucaugugaaaacugcAu................ 2 1 Mm3
......................................................................ugagaucaugGgaaaacugcuu................ 10 1 Mm3
......................................................................ugagaucaugugaaaacugAuu................ 3 1 Mm3
......................................................................ugagauAaugugaaaacugcuu................ 1 1 Mm3
......................................................................ugagaNcaugugaaaacugcuu................ 2 1 Mm3
......................................................................ugagaucaugugaaUacugcuu................ 1 1 Mm3
......................................................................ugagaucaugugaaaCcugcuu................ 3 1 Mm3
......................................................................ugagaucaugugaGaacugcuuu............... 1 1 Mm3
......................................................................ugagaucUugugaaaacugcuuu............... 1 1 Mm3
......................................................................ugagaucaugugaaaacugcCuu............... 1 1 Mm3
......................................................................ugagaucaugugaaaacugcuuG............... 1 1 Mm3
......................................................................ugagaucaugugaaaacugcGuu............... 1 1 Mm3
......................................................................ugagaucaugAgaaaacugcuuu............... 7 1 Mm3
......................................................................ugagaucaugugaaaacugcuuC............... 2 1 Mm3
......................................................................uNagaucaugugaaaacugcuuu............... 1 1 Mm3
......................................................................ugagaucaugugaaaacAgcuuu............... 8 1 Mm3
......................................................................ugagaucaugugaaaaAugcuuu............... 6 1 Mm3
......................................................................ugagaucaugGgaaaacugcuuu............... 2 1 Mm3
......................................................................ugagGucaugugaaaacugcuuu............... 1 1 Mm3
......................................................................ugagaucaugugaaaacCgcuuu............... 8 1 Mm3
......................................................................ugagaucaugugaaaaUugcuuu............... 4 1 Mm3
......................................................................ugagaucaugugaaaacugcuGu............... 1 1 Mm3
......................................................................uAagaucaugugaaaacugcuuu............... 1 1 Mm3
......................................................................ugagaucaugugaaaacugcuuu............... 1662 0 Mm3
......................................................................ugagaucaugCgaaaacugcuuu............... 1 1 Mm3
......................................................................ugagaucaugugaaaacugcuuA............... 122 1 Mm3
......................................................................ugagCucaugugaaaacugcuuu............... 1 1 Mm3
......................................................................ugagaucaugugaaaacGgcuuu............... 10 1 Mm3
......................................................................ugagaucaugugaaaacugcuAu............... 2 1 Mm3
......................................................................ugagaucaugugaaUacugcuuu............... 1 1 Mm3
......................................................................ugagaucaugugaaaacugcuuAu.............. 7 1 Mm3
......................................................................ugagaucaugugaaaacAgcuuuu.............. 3 1 Mm3
......................................................................ugagaucaugugaaaacGgcuuuu.............. 2 1 Mm3
......................................................................ugagaucaCgugaaaacugcuuuu.............. 1 1 Mm3
......................................................................ugGgaucaugugaaaacugcuuuu.............. 1 1 Mm3
......................................................................ugagaucaugugaaaacugcuuuA.............. 9 1 Mm3
......................................................................ugagaucaugugaaaacCgcuuuu.............. 1 1 Mm3
......................................................................ugagaucaugugaaaacugcuuuu.............. 213 0 Mm3
......................................................................ugUgaucaugugaaaacugcuuuu.............. 1 1 Mm3
......................................................................ugagaucaugugaaaacugcuuuuU............. 26 1 Mm3
......................................................................ugagaucaugugaaaacugcuuuuCu............ 1 1 Mm3
......................................................................ugagaucaugugaaaacugcuuuuUu............ 5 1 Mm3
.......................................................................gagaucaugugaaaacugc.................. 1 0 Mm3
.......................................................................gagaucaugugaaaacugcu................. 2 0 Mm3
.......................................................................gagaucaugugaaaacugcuu................ 6 0 Mm3
.......................................................................gagaucaugugaaaacugcuuu............... 2 0 Mm3
........................................................................agaucaugugaaaacugcuu................ 1 0 Mm3

..............................ccgguuuucaCaaugauu............................................................ 2 1 Fm1
..............................ccgguuuucauaaugauuuu.......................................................... 4 0 Fm1
..............................ccgguuuucauaaugauuuuac........................................................ 5 0 Fm1
..............................ccgguuuucauaaugauGuuac........................................................ 1 1 Fm1
..............................ccgguuuucaCaaugauuuuac........................................................ 12 1 Fm1
..............................ccgguuuucaCaaugauuuuaca....................................................... 7 1 Fm1
..............................ccgguuuucauaaugauCuuaca....................................................... 1 1 Fm1
..............................Ucgguuuucauaaugauuuuaca....................................................... 1 1 Fm1
..............................ccgguuuucauaaugauuuuaca....................................................... 25 0 Fm1
..............................ccgguuuucauUaugauuuuaca....................................................... 1 1 Fm1
..............................ccgguuuucaCaaugauuuuacaga..................................................... 17 1 Fm1
..............................ccgguuuucauaaugauuuuacaga..................................................... 19 0 Fm1
..............................ccgguuuucauaaugauCuuacaga..................................................... 1 1 Fm1
....................................................................ucugagaucaugugaaaacu.................... 2 0 Fm1
```

Star Mature

ggacauggagcucuaccauaaaagacgaaaccgguuuucauaaugauuuuacagauugauuuugaaacucugagaucaugugaaaacugcuuuugucgacagugauca			
....ucugagaucaugugaaaacugc..................	3	0	Fm1
....cugagaucaugugaaaacugcu.................	12	0	Fm1
....cugagaucaugugaaaaAugcu.................	1	1	Fm1
....cugagaucaugugaaaacugcuu................	9	0	Fm1
....ugagaucaugugaaaacu....................	328	0	Fm1
....ugagaucaugugaaUacu....................	1	1	Fm1
....ugagaucaugugaaaacC....................	1	1	Fm1
....ugagaucaugugaaaacG....................	2	1	Fm1
....ugagaucaugugaaGacu....................	1	1	Fm1
....ugagaucaugugaaaaAu....................	1	1	Fm1
....ugagaucauUugaaaacu....................	1	1	Fm1
....ugagaucaugugaGaacu....................	1	1	Fm1
....ugagaucaugugaaaacA....................	3	1	Fm1
....ugagaucaugugaaaaUu....................	1	1	Fm1
....ugagaucaugugaaaacuA...................	40	1	Fm1
....ugagaucaugugaaaacGg...................	12	1	Fm1
....ugagaucaugugaaaacuU...................	15	1	Fm1
....ugaCaucaugugaaaacug...................	1	1	Fm1
....ugagaucaugAgaaaacug...................	3	1	Fm1
....ugagaucaugugaaaaUug...................	3	1	Fm1
....ugagaucaugugaaaacug...................	1196	0	Fm1
....ugagaucaugugaaaacuC...................	1	1	Fm1
....ugGgaucaugugaaaacug...................	1	1	Fm1
....ugagaucaugugaaaacCg...................	12	1	Fm1
....ugagaucaugugaaaaAug...................	5	1	Fm1
....ugagaucauUugaaaacug...................	1	1	Fm1
....ugagaucauCugaaaacug...................	1	1	Fm1
....ugagaucaugugaCaacug...................	1	1	Fm1
....ugagaucaugugaaaacAg...................	5	1	Fm1
....ugagaucaugAgaaaacugc..................	1	1	Fm1
....ugagaucaugugaaaacCgc..................	6	1	Fm1
....Ggagaucaugugaaaacugc..................	1	1	Fm1
....ugagaucaugugaaaacugG..................	2	1	Fm1
....ugagaucaugGgaaaacugc..................	1	1	Fm1
....ugagaucaugugaaaaAugc..................	6	1	Fm1
....ugagaucaugugaaaacugc..................	1117	0	Fm1
....ugagaucaugugaaaacGgc..................	8	1	Fm1
....ugagaucaugugaaaacAgc..................	9	1	Fm1
....ugagaucaugugaaaaGugc..................	2	1	Fm1
....ugagaucaugugGaaacugc..................	1	1	Fm1
....ugagaucaugugCaaacugc..................	1	1	Fm1
....ugagaucaugugaaaaUugc..................	1	1	Fm1
....ugagaucaugugaaaacugU..................	41	1	Fm1
....ugagaucaugugaaUacugcu.................	1	1	Fm1
....Cgagaucaugugaaaacugcu.................	1	1	Fm1
....ugUgaucaugugaaaacugcu.................	2	1	Fm1
....ugagaucaugugaaaacugAu.................	2	1	Fm1
....ugagaucaugCgaaaacugcu.................	7	1	Fm1
....ugagaucaugugaaaacAgcu.................	11	1	Fm1
....ugagaucaugGgaaaacugcu.................	3	1	Fm1
....ugaAaucaugugaaaacugcu.................	1	1	Fm1
....ugagaucaugugaaaacuAcu.................	1	1	Fm1
....ugagaucaugugaaaacugcA.................	9	1	Fm1
....ugagaucaugugGaaacugcu.................	2	1	Fm1
....ugagauAaugugaaaacugcu.................	1	1	Fm1
....ugagaucaugugaaGacugcu.................	1	1	Fm1
....uAagaucaugugaaaacugcu.................	2	1	Fm1
....ugagaucaugugaaaacCgcu.................	24	1	Fm1
....ugagaucaugugaaaaGugcu.................	6	1	Fm1
....ugagaucaugugaaaacuUcu.................	1	1	Fm1
....Ggagaucaugugaaaacugcu.................	1	1	Fm1
....ugagaucaugugaaaacugcC.................	2	1	Fm1
....ugagaucaugAgaaaacugcu.................	7	1	Fm1
....ugagaucaugugUaaacugcu.................	1	1	Fm1
....ugagaucaCgugaaaacugcu.................	2	1	Fm1
....ugGgaucaugugaaaacugcu.................	2	1	Fm1
....ugagaucaugugaaaacGgcu.................	18	1	Fm1
....ugCgaucaugugaaaacugcu.................	1	1	Fm1
....ugagaucaugugaaaaAugcu.................	15	1	Fm1
....ugagaucaugugaaaacugcG.................	12	1	Fm1

Star   Mature

```
ggacauggagcucuaccauaaaagacgaaaccgguuuucauaaugauuuuacagauugauuuugaaacucugagaucaugugaaaacugcuuuugucgacagugauca
......................................................................ugagaGcaugugaaaacugcu.................   1      1   Fm1
......................................................................ugagaucaugugaaaacugUu.................   16     1   Fm1
......................................................................ugagaucaugugaCaacugcu.................   1      1   Fm1
......................................................................ugagaucauAugaaaacugcu.................   1      1   Fm1
......................................................................ugagaucaugugaaaacugcu.................   3519   0   Fm1
......................................................................ugagaucaugugaaaaUugcu.................   9      1   Fm1
......................................................................ugagaucauguAaaaacugcu.................   1      1   Fm1
......................................................................ugagaNcaugugaaaacugcu.................   1      1   Fm1
......................................................................ugagaucauUugaaaacugcu.................   1      1   Fm1
......................................................................ugagCucaugugaaaacugcu.................   2      1   Fm1
......................................................................Ngagaucaugugaaaacugcu.................   2      1   Fm1
......................................................................ugagaucaugugCaaacugcuu................   2      1   Fm1
......................................................................ugagaucaugugaaGacugcuu................   1      1   Fm1
......................................................................ugagaucauCugaaaacugcuu................   2      1   Fm1
......................................................................ugagaucaugCgaaaacugcuu................   14     1   Fm1
......................................................................uUagaucaugugaaaacugcuu................   5      1   Fm1
......................................................................ugagaucaugugUaaacugcuu................   1      1   Fm1
......................................................................ugUgaucaugugaaaacugcuu................   2      1   Fm1
......................................................................ugagaucauUugaaaacugcuu................   1      1   Fm1
......................................................................ugagaucaugugaaaacugcuA................   3      1   Fm1
......................................................................ugagaucaGgugaaaacugcuu................   1      1   Fm1
......................................................................Cgagaucaugugaaaacugcuu................   5      1   Fm1
......................................................................ugagaCcaugugaaaacugcuu................   2      1   Fm1
......................................................................Ggagaucaugugaaaacugcuu................   1      1   Fm1
......................................................................ugagauGaugugaaaacugcuu................   3      1   Fm1
......................................................................ugCgaucaugugaaaacugcuu................   1      1   Fm1
......................................................................ugagUucaugugaaaacugcuu................   1      1   Fm1
......................................................................ugagaucUugugaaaacugcuu................   7      1   Fm1
......................................................................ugagaucaugugaaaacGgcuu................   76     1   Fm1
......................................................................ugagaucaugGgaaaacugcuu................   16     1   Fm1
......................................................................Agagaucaugugaaaacugcuu................   4      1   Fm1
......................................................................ugagaucaugugaaaUcugcuu................   3      1   Fm1
......................................................................ugagaucaugugaaaaGugcuu................   32     1   Fm1
......................................................................ugagaucaAgugaaaacugcuu................   1      1   Fm1
......................................................................ugagaucaugugGaaacugcuu................   1      1   Fm1
......................................................................ugagaucaugugaaaacugUuu................   63     1   Fm1
......................................................................ugagaucGugugaaaacugcuu................   3      1   Fm1
......................................................................ugaAaucaugugaaaacugcuu................   1      1   Fm1
......................................................................ugagaucaugugaaaaAugcuu................   71     1   Fm1
......................................................................ugagaucaugugaaaacAgcuu................   85     1   Fm1
......................................................................uAagaucaugugaaaacugcuu................   9      1   Fm1
......................................................................ugagaucaugugaaaacuAcuu................   3      1   Fm1
......................................................................ugagaucaugugaaCacugcuu................   1      1   Fm1
......................................................................ugaCaucaugugaaaacugcuu................   2      1   Fm1
......................................................................ugagaucauAugaaaacugcuu................   2      1   Fm1
......................................................................ugagauUaugugaaaacugcuu................   7      1   Fm1
......................................................................ugagaucaugugaaaacugcuu................   17152  0   Fm1
......................................................................ugagauNaugugaaaacugcuu................   2      1   Fm1
......................................................................ugagaucaugugaaaacCgcuu................   97     1   Fm1
......................................................................ugagaucaugugaaaacugcCu................   5      1   Fm1
......................................................................ugagaucaugugaaaacugcuC................   2      1   Fm1
......................................................................uCagaucaugugaaaacugcuu................   3      1   Fm1
......................................................................ugagaucaugugaGaacugcuu................   4      1   Fm1
......................................................................ugagaucaugugaaaacugcGu................   3      1   Fm1
......................................................................ugagaucaugugaaaacugGuu................   2      1   Fm1
......................................................................ugagaucaugugaUaacugcuu................   2      1   Fm1
......................................................................ugGgaucaugugaaaacugcuu................   9      1   Fm1
......................................................................ugagaNcaugugaaaacugcuu................   2      1   Fm1
......................................................................ugagaucaugugaCaacugcuu................   4      1   Fm1
......................................................................ugagaucaugugaaaacugAuu................   6      1   Fm1
......................................................................ugagaucaugugaaaaUugcuu................   38     1   Fm1
......................................................................Ngagaucaugugaaaacugcuu................   9      1   Fm1
......................................................................ugagaucaugAgaaaacugcuu................   31     1   Fm1
......................................................................ugagaucaugugaaaacugcuG................   7      1   Fm1
......................................................................ugagCucaugugaaaacugcuu................   8      1   Fm1
......................................................................ugagaucaCgugaaaacugcuu................   3      1   Fm1
......................................................................ugagaucauguUaaaacugcuu................   1      1   Fm1
......................................................................ugagauAaugugaaaacugcuu................   1      1   Fm1
......................................................................ugagGucaugugaaaacugcuu................   3      1   Fm1
......................................................................ugagaucaugGgaaaacugcuuu...............   2      1   Fm1
```

```
                              Star                                    Mature
ggacauggagcucuaccauaaaagacgaaaccgguuuucauaaugauuuuacagauugauuuugaaacucugagaucaugugaaaacugcuuuugucgacagugauca
......................................................................ugagaucaugugaaaacugcuuA...............   92     1     Fm1
......................................................................ugagaucaugugaaaacugcuuG...............   6      1     Fm1
......................................................................ugagaucaugAgaaaacugcuuu...............   7      1     Fm1
......................................................................uAagaucaugugaaaacugcuuu...............   1      1     Fm1
......................................................................ugGgaucaugugaaaacugcuuu...............   1      1     Fm1
......................................................................ugCgaucaugugaaaacugcuuu...............   1      1     Fm1
......................................................................ugagaucaugugaGaacugcuuu...............   1      1     Fm1
......................................................................Ngagaucaugugaaaacugcuuu...............   2      1     Fm1
......................................................................ugagaucaugugaaaaGugcuuu...............   3      1     Fm1
......................................................................ugagaucaugugaaaaUugcuuu...............   7      1     Fm1
......................................................................ugagaucaugugaaaaAugcuuu...............   2      1     Fm1
......................................................................ugagaucaugugaaaacugcAuu...............   1      1     Fm1
......................................................................ugagaucaugugCaaacugcuuu...............   1      1     Fm1
......................................................................ugagauUaugugaaaacugcuuu...............   2      1     Fm1
......................................................................ugagaucaugugaaaacCgcuuu...............   13     1     Fm1
......................................................................Cgagaucaugugaaaacugcuuu...............   2      1     Fm1
......................................................................ugaUaucaugugaaaacugcuuu...............   1      1     Fm1
......................................................................ugagaucaugugaaaacuUcuuu...............   1      1     Fm1
......................................................................ugagauAaugugaaaacugcuuu...............   1      1     Fm1
......................................................................ugagaucaugugaaaacugcuuC...............   5      1     Fm1
......................................................................ugagaucaugugaaaacAgcuuu...............   9      1     Fm1
......................................................................ugagaucaugugaaaacGgcuuu...............   5      1     Fm1
......................................................................ugagaucaugugaaaacugcuuu...............   1835   0     Fm1
......................................................................ugagaucaugugaaaacugUuuu...............   8      1     Fm1
......................................................................ugagaucaugugaaaacugcCuu...............   1      1     Fm1
......................................................................ugagaucaugugaaaacugcuCu...............   1      1     Fm1
......................................................................ugagaucaugCgaaaacugcuuu...............   1      1     Fm1
......................................................................ugagaucaugugaaaacGgcuuuu..............   2      1     Fm1
......................................................................ugagaucaugugaaaacugcuuAu..............   21     1     Fm1
......................................................................ugagaucaugugaaaaAugcuuuu..............   2      1     Fm1
......................................................................ugagaucaugugaaaaGugcuuuu..............   1      1     Fm1
......................................................................ugagauAaugugaaaacugcuuuu..............   1      1     Fm1
......................................................................ugagaucaugugaaaacugcuuuA..............   14     1     Fm1
......................................................................ugagaucaugugaaaacugcuuCu..............   4      1     Fm1
......................................................................ugagaucauUugaaaacugcuuuu..............   1      1     Fm1
......................................................................ugagaucaugugaaaacugcuuuu..............   392    0     Fm1
......................................................................ugagaucaugugaaaacCgcuuuu..............   2      1     Fm1
......................................................................ugagaucaugugaaaacAgcuuuu..............   1      1     Fm1
......................................................................ugagaucaugugaaaacugcuuuuU.............   21     1     Fm1
.......................................................................gagaucaugugaaaacugcu.................   3      0     Fm1
.......................................................................gagaucaugugaaaacugcuu................   7      0     Fm1
.......................................................................gagaucaugugaaaacugcuuu...............   3      0     Fm1
.......................................................................gagaucaugugaaaacugcuuuA..............   1      1     Fm1
.......................................................................gagaucaugugaaaacugcuuuu..............   2      0     Fm1
.........................................................................gaucaugugaaaacugcuu................   3      0     Fm1

..............................ccgguuuucauaaugauu............................................................   6      0     Fm2
..............................ccgguuuucauaaugauuuuac........................................................   38     0     Fm2
..............................ccgguuuucauaaugauAuuaca.......................................................   1      1     Fm2
..............................ccgguuuucauaaugUuuuuaca.......................................................   1      1     Fm2
..............................ccgguuuucauaaugauuuuUca.......................................................   1      1     Fm2
..............................ccgguuuucauaaugauuuuaca.......................................................   132    0     Fm2
..............................ccgguuuucauaaugauGuuacaga.....................................................   1      1     Fm2
..............................ccgguuuucauaaugauuuuacagG.....................................................   1      1     Fm2
..............................ccgguuuucauaaugaGuuuacaga.....................................................   1      1     Fm2
..............................ccgguuuucauaaugauuuuacaga.....................................................   66     0     Fm2
..............................ccgguuuucauaaugauCuuacaga.....................................................   1      1     Fm2
..............................ccgguuuucauaaugaAuuuacaga.....................................................   1      1     Fm2
.....................................................................cugagaucaugugaaaacugcG.................   1      1     Fm2
.....................................................................cugagaucaugugaaaacugcu.................   32     0     Fm2
......................................................................ugagaucaugugaaaacA....................   1      1     Fm2
......................................................................ugagaucaugugaaaacu....................   204    0     Fm2
......................................................................Ngagaucaugugaaaacu....................   1      1     Fm2
......................................................................ugagaucaugugaaaacG....................   1      1     Fm2
......................................................................ugaAaucaugugaaaacug...................   1      1     Fm2
......................................................................ugagaAcaugugaaaacug...................   1      1     Fm2
......................................................................ugagaucaugugaaaaUug...................   3      1     Fm2
......................................................................ugagaucaugGgaaaacug...................   1      1     Fm2
......................................................................ugagaucauguUaaaacug...................   1      1     Fm2
......................................................................ugagaucaugugaaaacAg...................   17     1     Fm2
```

Star Mature

```
ggacauggagcucuaccauaaaagacgaaaccgguuuucauaaugauuuuacagauugauuuugaaacucugagaucaugugaaaacugcuuuugucgacagugauca
......................................................................ugagaucaugugUaaacug...................   1      1   Fm2
......................................................................Ngagaucaugugaaaacug...................   1      1   Fm2
......................................................................uUagaucaugugaaaacug...................   1      1   Fm2
......................................................................uAagaucaugugaaaacug...................   2      1   Fm2
......................................................................ugagaucaugugaaaacuA...................   11     1   Fm2
......................................................................ugagGucaugugaaaacug...................   1      1   Fm2
......................................................................ugagaucaugugaaaaAug...................   11     1   Fm2
......................................................................ugagaucaugAgaaaacug...................   6      1   Fm2
......................................................................ugagaucaugugaaaacuU...................   16     1   Fm2
......................................................................ugagaucaugugaaaacCg...................   8      1   Fm2
......................................................................ugagUucaugugaaaacug...................   1      1   Fm2
......................................................................ugagaucaugugaaCacug...................   1      1   Fm2
......................................................................ugagaucaugugaaaacGg...................   31     1   Fm2
......................................................................ugagaucaugugaaaacug...................   2387   0   Fm2
......................................................................Cgagaucaugugaaaacug...................   1      1   Fm2
......................................................................ugagaucaugugaGaacug...................   1      1   Fm2
......................................................................ugagaucauguAaaaacug...................   1      1   Fm2
......................................................................ugagaucaugCgaaaacug...................   4      1   Fm2
......................................................................ugagaucaugugaaaaGugc..................   4      1   Fm2
......................................................................ugagaucaugugaaaaAugc..................   9      1   Fm2
......................................................................ugagaucaugugaaaacugG..................   1      1   Fm2
......................................................................ugagaucaugugaUaacugc..................   1      1   Fm2
......................................................................uUagaucaugugaaaacugc..................   2      1   Fm2
......................................................................ugagGucaugugaaaacugc..................   1      1   Fm2
......................................................................ugagaucUugugaaaacugc..................   1      1   Fm2
......................................................................ugagaucaugAgaaaacugc..................   8      1   Fm2
......................................................................ugagaucaugugaaaacugU..................   12     1   Fm2
......................................................................ugagaucaugugaaaacAgc..................   14     1   Fm2
......................................................................ugagaucaugugaaaacCgc..................   8      1   Fm2
......................................................................ugagaucaugugaaaUcugc..................   2      1   Fm2
......................................................................ugagaucaugGgaaaacugc..................   1      1   Fm2
......................................................................ugagaucaugugaaaacuUc..................   1      1   Fm2
......................................................................ugagaucaugugGaaacugc..................   1      1   Fm2
......................................................................ugagaucaugugaaaacGgc..................   15     1   Fm2
......................................................................ugagaucaugCgaaaacugc..................   2      1   Fm2
......................................................................ugagaucaugugaaaaUugc..................   2      1   Fm2
......................................................................ugUgaucaugugaaaacugc..................   1      1   Fm2
......................................................................ugagaucaugugaaaacugc..................   1846   0   Fm2
......................................................................ugagaCcaugugaaaacugc..................   1      1   Fm2
......................................................................ugagaucaugugaaaacGgcu.................   75     1   Fm2
......................................................................ugagaucaugugaaaacCgcu.................   75     1   Fm2
......................................................................ugagaucauguCaaaacugcu.................   2      1   Fm2
......................................................................ugagaucaCgugaaaacugcu.................   6      1   Fm2
......................................................................ugagaucaugugaaaacuUcu.................   1      1   Fm2
......................................................................ugagaucauAugaaaacugcu.................   3      1   Fm2
......................................................................ugagGucaugugaaaacugcu.................   5      1   Fm2
......................................................................ugagaucauguAaaaacugcu.................   1      1   Fm2
......................................................................ugagaucaugugaaaacugcu.................   14644  0   Fm2
......................................................................ugagaucaugugGaaacugcu.................   4      1   Fm2
......................................................................uCagaucaugugaaaacugcu.................   2      1   Fm2
......................................................................ugagaucaugugUaaacugcu.................   2      1   Fm2
......................................................................ugagaucaugugaaaaGugcu.................   23     1   Fm2
......................................................................ugagaCcaugugaaaacugcu.................   3      1   Fm2
......................................................................ugagaucaugugaaaGcugcu.................   3      1   Fm2
......................................................................uUagaucaugugaaaacugcu.................   1      1   Fm2
......................................................................ugagaucaugugaaaaAugcu.................   68     1   Fm2
......................................................................ugagaucaugugaCaacugcu.................   2      1   Fm2
......................................................................ugagaucUugugaaaacugcu.................   2      1   Fm2
......................................................................ugaCaucaugugaaaacugcu.................   3      1   Fm2
......................................................................ugagauUaugugaaaacugcu.................   7      1   Fm2
......................................................................ugagaucCugugaaaacugcu.................   1      1   Fm2
......................................................................ugagaucaugGgaaaacugcu.................   13     1   Fm2
......................................................................uAagaucaugugaaaacugcu.................   6      1   Fm2
......................................................................ugagaucaugugaaaacugcA.................   34     1   Fm2
......................................................................ugagaucaugugaaaacugcC.................   1      1   Fm2
......................................................................ugagaucaugugaaaacAgcu.................   63     1   Fm2
......................................................................ugagaucaugugaUaacugcu.................   1      1   Fm2
......................................................................ugagaNcaugugaaaacugcu.................   2      1   Fm2
......................................................................ugagaucaugugaaaacuAcu.................   1      1   Fm2
......................................................................ugagaucauguUaaaacugcu.................   1      1   Fm2
```

```
                                            Star                                    Mature
ggacauggagcucuaccauaaaaagacgaaaccgguuuucauaaugauuuuacagauugauuuugaaacucugagaucaugugaaaacugcuuuugucgacagugauca
.......................................................................ugUgaucaugugaaaacugcu.................   2       1       Fm2
.......................................................................ugagauAaugugaaaacugcu.................   3       1       Fm2
.......................................................................Ngagaucaugugaaaacugcu.................   5       1       Fm2
.......................................................................ugagaucaugugaaaaUugcu.................   32      1       Fm2
.......................................................................ugagaucaugugaaaacugAu.................   2       1       Fm2
.......................................................................ugagaucaugugaaaacugcG.................   49      1       Fm2
.......................................................................ugagaucaugAgaaaacugcu.................   27      1       Fm2
.......................................................................ugagCucaugugaaaacugcu.................   3       1       Fm2
.......................................................................ugagaAcaugugaaaacugcu.................   5       1       Fm2
.......................................................................ugagaucaugCgaaaacugcu.................   17      1       Fm2
.......................................................................ugagaucaugugaGaacugcu.................   3       1       Fm2
.......................................................................Cgagaucaugugaaaacugcu.................   2       1       Fm2
.......................................................................Agagaucaugugaaaacugcu.................   1       1       Fm2
.......................................................................ugagaucGugugaaaacugcu.................   1       1       Fm2
.......................................................................ugagaucaugugaaaUcugcu.................   3       1       Fm2
.......................................................................ugGgaucaugugaaaacugcu.................   2       1       Fm2
.......................................................................ugagaucaugugaaaacuCcu.................   1       1       Fm2
.......................................................................ugagaucaAgugaaaacugcu.................   1       1       Fm2
.......................................................................ugagaucaugugaaaacugUu.................   7       1       Fm2
.......................................................................ugagaucaugugaaaCcugcu.................   2       1       Fm2
.......................................................................ugagUucaugugaaaacugcu.................   3       1       Fm2
.......................................................................ugagaucauCugaaaacugcuu................   3       1       Fm2
.......................................................................ugagaucaugAgaaaacugcuu................   46      1       Fm2
.......................................................................ugaCaucaugugaaaacugcuu................   1       1       Fm2
.......................................................................ugagaucGugugaaaacugcuu................   3       1       Fm2
.......................................................................ugagaucaugugGaaacugcuu................   4       1       Fm2
.......................................................................Ngagaucaugugaaaacugcuu................   10      1       Fm2
.......................................................................ugagaucaugugaaaacugcuA................   30      1       Fm2
.......................................................................ugagaucaugugaaaacugcCu................   2       1       Fm2
.......................................................................ugagaucaugugaaaacuUcuu................   2       1       Fm2
.......................................................................ugagaucauUugaaaacugcuu................   2       1       Fm2
.......................................................................ugagaucaugugaaaacCgcuu................   153     1       Fm2
.......................................................................ugaUaucaugugaaaacugcuu................   1       1       Fm2
.......................................................................ugagaucaugugaaaacGgcuu................   124     1       Fm2
.......................................................................ugagauAaugugaaaacugcuu................   3       1       Fm2
.......................................................................ugNgaucaugugaaaacugcuu................   1       1       Fm2
.......................................................................ugagaucaugugaaaGcugcuu................   2       1       Fm2
.......................................................................ugagaAcaugugaaaacugcuu................   2       1       Fm2
.......................................................................ugagaucaugugUaaacugcuu................   6       1       Fm2
.......................................................................ugagaucaugugaaaacugAuu................   3       1       Fm2
.......................................................................Cgagaucaugugaaaacugcuu................   6       1       Fm2
.......................................................................ugagaucaugugaaaacuAcuu................   3       1       Fm2
.......................................................................ugagUucaugugaaaacugcuu................   1       1       Fm2
.......................................................................ugagaucauAugaaaacugcuu................   2       1       Fm2
.......................................................................ugagaucauguCaaaacugcuu................   2       1       Fm2
.......................................................................ugagaucaugugaaaacugcGu................   1       1       Fm2
.......................................................................ugaAaucaugugaaaacugcuu................   3       1       Fm2
.......................................................................ugagaNcaugugaaaacugcuu................   3       1       Fm2
.......................................................................Agagaucaugugaaaacugcuu................   3       1       Fm2
.......................................................................ugagaCcaugugaaaacugcuu................   1       1       Fm2
.......................................................................ugagauUaugugaaaacugcuu................   10      1       Fm2
.......................................................................ugagaucaugugaaUacugcuu................   4       1       Fm2
.......................................................................ugagaucaugugaaaacugcuC................   2       1       Fm2
.......................................................................ugagaucaugugaaaaAugcuu................   89      1       Fm2
.......................................................................ugagaucaugugaGaacugcuu................   1       1       Fm2
.......................................................................uUagaucaugugaaaacugcuu................   3       1       Fm2
.......................................................................ugagaucaugugaCaacugcuu................   2       1       Fm2
.......................................................................ugagaucaugugaaaacugcuG................   22      1       Fm2
.......................................................................ugagaucaugugaaaacugcuu................   26527   0       Fm2
.......................................................................ugagaucaCgugaaaacugcuu................   4       1       Fm2
.......................................................................uNagaucaugugaaaacugcuu................   1       1       Fm2
.......................................................................ugUgaucaugugaaaacugcuu................   5       1       Fm2
.......................................................................ugagaucauguUaaaacugcuu................   2       1       Fm2
.......................................................................ugagauNaugugaaaacugcuu................   3       1       Fm2
.......................................................................ugagaucaugugaaaacAgcuu................   149     1       Fm2
.......................................................................ugagaucaugugaaaUcugcuu................   5       1       Fm2
.......................................................................ugagauGaugugaaaacugcuu................   2       1       Fm2
.......................................................................ugGgaucaugugaaaacugcuu................   22      1       Fm2
.......................................................................ugagaucUugugaaaacugcuu................   7       1       Fm2
.......................................................................ugagaucaugugaUaacugcuu................   2       1       Fm2
```

Star Mature

```
ggacauggagcucuaccauaaaagacgaaaccgguuuucauaaugauuuuacagauugauuuugaaacucugagaucaugugaaaacugcuuuugucgacagugauca
......................................................................ugagaucauguAaaaacugcuu................   2     1   Fm2
......................................................................uCagaucaugugaaaacugcuu................   1     1   Fm2
......................................................................ugagaucaugugCaaacugcuu................   3     1   Fm2
......................................................................ugagaucaugCgaaaacugcuu................   28    1   Fm2
......................................................................ugagaucaugugaaaaGugcuu................   37    1   Fm2
......................................................................ugagaucaugugaaaCcugcuu................   2     1   Fm2
......................................................................ugagCucaugugaaaacugcuu................   10    1   Fm2
......................................................................ugagaucaugGgaaaacugcuu................   29    1   Fm2
......................................................................ugagaucaugugaaaaUugcuu................   44    1   Fm2
......................................................................ugagaucaugugaaaacugUuu................   36    1   Fm2
......................................................................ugagaucaugugaaGacugcuu................   6     1   Fm2
......................................................................ugagaucaAgugaaaacugcuu................   2     1   Fm2
......................................................................uAagaucaugugaaaacugcuu................   9     1   Fm2
......................................................................ugagGucaugugaaaacugcuu................   5     1   Fm2
......................................................................ugagCucaugugaaaacugcuuu...............   1     1   Fm2
......................................................................ugagaucaugugaaaacCgcuuu...............   23    1   Fm2
......................................................................ugagaucauguUaaaacugcuuu...............   1     1   Fm2
......................................................................ugaUaucaugugaaaacugcuuu...............   1     1   Fm2
......................................................................ugagaucaugugUaaacugcuuu...............   1     1   Fm2
......................................................................ugagaucaugugaaaaAugcuuu...............   16    1   Fm2
......................................................................ugagaucaugugaaaacugUuuu...............   7     1   Fm2
......................................................................Ngagaucaugugaaaacugcuuu...............   2     1   Fm2
......................................................................ugagUucaugugaaaacugcuuu...............   1     1   Fm2
......................................................................ugagaucauguAaaaacugcuuu...............   1     1   Fm2
......................................................................Cgagaucaugugaaaacugcuuu...............   2     1   Fm2
......................................................................ugagaucaugugaaaacugcuuG...............   22    1   Fm2
......................................................................ugagaucaugugaaaacugcuuu...............   3250  0   Fm2
......................................................................ugCgaucaugugaaaacugcuuu...............   1     1   Fm2
......................................................................ugagaucaugugaaaacGgcuuu...............   12    1   Fm2
......................................................................ugagaucaugAgaaaacugcuuu...............   3     1   Fm2
......................................................................ugagaucaugCgaaaacugcuuu...............   5     1   Fm2
......................................................................ugagaucaugGgaaaacugcuuu...............   7     1   Fm2
......................................................................ugagaucaugugaaaaUugcuuu...............   6     1   Fm2
......................................................................uAagaucaugugaaaacugcuuu...............   3     1   Fm2
......................................................................ugagaucGugugaaaacugcuuu...............   1     1   Fm2
......................................................................ugagaucCugugaaaacugcuuu...............   1     1   Fm2
......................................................................ugaAaucaugugaaaacugcuuu...............   1     1   Fm2
......................................................................ugagaucaugugaaaaGugcuuu...............   5     1   Fm2
......................................................................ugagaucaugugaaaacugcuuC...............   22    1   Fm2
......................................................................ugagaucaugugaaaacugcCuu...............   1     1   Fm2
......................................................................ugagaucaugugaaaacugcuAu...............   1     1   Fm2
......................................................................ugGgaucaugugaaaacugcuuu...............   4     1   Fm2
......................................................................ugagaucaugugaaaacugcuuA...............   250   1   Fm2
......................................................................ugagaucaugugaaaacAgcuuu...............   22    1   Fm2
......................................................................ugUgaucaugugaaaacugcuuu...............   1     1   Fm2
......................................................................ugGgaucaugugaaaacugcuuuu..............   1     1   Fm2
......................................................................ugagaucaugugaaaacAgcuuuu..............   3     1   Fm2
......................................................................ugagaucaugugaaaacugcuuAu..............   46    1   Fm2
......................................................................ugagCucaugugaaaacugcuuuu..............   2     1   Fm2
......................................................................ugagaucaugAgaaaacugcuuuu..............   2     1   Fm2
......................................................................ugagaucaugugaaaacugcuuuu..............   1101  0   Fm2
......................................................................ugagaucaugugaaaacGgcuuuu..............   7     1   Fm2
......................................................................ugagaucaugugaaaacCgcuuuu..............   5     1   Fm2
......................................................................ugagaucaugugaaaacugcCuuu..............   1     1   Fm2
......................................................................uAagaucaugugaaaacugcuuuu..............   1     1   Fm2
......................................................................ugagaucaugugaaaacugcuuGu..............   41    1   Fm2
......................................................................Agagaucaugugaaaacugcuuuu..............   1     1   Fm2
......................................................................ugagaucaugugaaaaUugcuuuu..............   5     1   Fm2
......................................................................ugagaucaugugaaaacugcuuuA..............   22    1   Fm2
......................................................................ugagaucUugugaaaacugcuuuu..............   1     1   Fm2
......................................................................ugagaNcaugugaaaacugcuuuu..............   1     1   Fm2
......................................................................ugagaucaugugaaaaAugcuuuu..............   6     1   Fm2
......................................................................ugagaucaugugaaaacugcuuuuU.............   69    1   Fm2
.......................................................................gagaucaugugaaaacugcuu................   4     0   Fm2
............................................................................caugugaaaacugcuuuugucgaca.......   13    0   Fm2

.....................................................................cugagaucaugugaaaacu....................   1     0   Fm3
.....................................................................cugagaucaugugaaaacugcu.................   7     0   Fm3
.....................................................................cugagaucaugugaaaaGugcuu................   1     1   Fm3
......................................................................Cgagaucaugugaaaacu....................   1     1   Fm3
```

```
                              Star                                      Mature
ggacauggagcucuaccauaaaagacgaaaccgguuuucauaaugauuuuacagauugauuuugaaacucugagaucaugugaaaacugcuuuugucgacagugauca
......................................................................ugagaucaugugUaaacu....................   1      1      Fm3
......................................................................uUagaucaugugaaaacu....................   1      1      Fm3
......................................................................ugagaucaugugaaaacC....................   3      1      Fm3
......................................................................ugagaucaugugaaaaAu....................   7      1      Fm3
......................................................................ugagaucaugugaaaacG....................   10     1      Fm3
......................................................................ugaCaucaugugaaaacu....................   1      1      Fm3
......................................................................ugagaucaugugaaaacu....................   1003   0      Fm3
......................................................................ugagauAaugugaaaacu....................   1      1      Fm3
......................................................................ugUgaucaugugaaaacu....................   1      1      Fm3
......................................................................ugagaucaugAgaaaacu....................   2      1      Fm3
......................................................................ugaAaucaugugaaaacu....................   1      1      Fm3
......................................................................ugagaucUugugaaaacu....................   1      1      Fm3
......................................................................ugagaucaugugaaaacA....................   6      1      Fm3
......................................................................ugagaucaugugGaaacu....................   1      1      Fm3
......................................................................ugagaucaugCgaaaacu....................   1      1      Fm3
......................................................................ugagaucaugugaaaacN....................   1      1      Fm3
......................................................................ugagaucaugugaaaacAg...................   11     1      Fm3
......................................................................ugagaucauUugaaaacug...................   2      1      Fm3
......................................................................ugagaucaugugaUaacug...................   1      1      Fm3
......................................................................ugUgaucaugugaaaacug...................   1      1      Fm3
......................................................................ugagaCcaugugaaaacug...................   1      1      Fm3
......................................................................ugagaucaugugaaaaAug...................   4      1      Fm3
......................................................................ugGgaucaugugaaaacug...................   1      1      Fm3
......................................................................Ngagaucaugugaaaacug...................   2      1      Fm3
......................................................................ugagaucaugugaaaacug...................   1635   0      Fm3
......................................................................ugagaucaugugaaaaUug...................   11     1      Fm3
......................................................................ugagaucauguAaaaacug...................   1      1      Fm3
......................................................................ugagaucaugGgaaaacug...................   1      1      Fm3
......................................................................ugagaucaugAgaaaacug...................   3      1      Fm3
......................................................................ugagaucaugugaaUacug...................   1      1      Fm3
......................................................................ugagaucaugugaaaacuU...................   18     1      Fm3
......................................................................ugaAaucaugugaaaacug...................   1      1      Fm3
......................................................................ugagaucUugugaaaacug...................   1      1      Fm3
......................................................................ugagaucaugugaaaacuA...................   126    1      Fm3
......................................................................ugagaucaugugaaaacCg...................   12     1      Fm3
......................................................................ugagaucaugCgaaaacug...................   5      1      Fm3
......................................................................ugagaucGugugaaaacug...................   1      1      Fm3
......................................................................ugagaucaugugaaaacGg...................   9      1      Fm3
......................................................................ugGgaucaugugaaaacugc..................   1      1      Fm3
......................................................................ugagaucaugugaaaacuAc..................   3      1      Fm3
......................................................................Ngagaucaugugaaaacugc..................   1      1      Fm3
......................................................................ugagaucaugugaaaaGugc..................   1      1      Fm3
......................................................................ugagaucaugugaaaacCgc..................   4      1      Fm3
......................................................................ugagaucaugugaaaaUugc..................   3      1      Fm3
......................................................................uCagaucaugugaaaacugc..................   1      1      Fm3
......................................................................uUagaucaugugaaaacugc..................   1      1      Fm3
......................................................................ugagaucaugugaaaaAugc..................   6      1      Fm3
......................................................................ugagaucaugugaaaacGgc..................   5      1      Fm3
......................................................................ugaAaucaugugaaaacugc..................   1      1      Fm3
......................................................................ugagaucaugugaaaacugU..................   24     1      Fm3
......................................................................ugagUucaugugaaaacugc..................   1      1      Fm3
......................................................................ugagauUaugugaaaacugc..................   1      1      Fm3
......................................................................ugagaucaugugaaaCcugc..................   1      1      Fm3
......................................................................ugaUaucaugugaaaacugc..................   1      1      Fm3
......................................................................ugagaucaugugaaaacAgc..................   5      1      Fm3
......................................................................ugagaucaugAgaaaacugc..................   1      1      Fm3
......................................................................ugagaucaugugaaaacugc..................   1013   0      Fm3
......................................................................ugagaucaugugaaaacuAcu.................   1      1      Fm3
......................................................................ugagaucaugugaaaacCgcu.................   32     1      Fm3
......................................................................ugagaucauguAaaaacugcu.................   1      1      Fm3
......................................................................ugagaucaugugaaaacAgcu.................   35     1      Fm3
......................................................................uUagaucaugugaaaacugcu.................   1      1      Fm3
......................................................................Ngagaucaugugaaaacugcu.................   2      1      Fm3
......................................................................ugagaucUugugaaaacugcu.................   2      1      Fm3
......................................................................ugagaucaugugaaaacugcC.................   2      1      Fm3
......................................................................ugagaucaugAgaaaacugcu.................   10     1      Fm3
......................................................................ugagaNcaugugaaaacugcu.................   1      1      Fm3
......................................................................ugagaucaugugaaaacGgcu.................   31     1      Fm3
......................................................................ugagaucauAugaaaacugcu.................   1      1      Fm3
......................................................................ugagaucaugugaaaUcugcu.................   1      1      Fm3
```

Star **Mature**

ggacauggagcucuaccauaaaaagacgaaaccgguuuucauaaugauuuuacagauugauuuugaaacucugagaucaugugaaaacugcuuuugucgacagugauca			
....ugagaucaugugaaaaUugcu.................	15	1	Fm3
....ugagaucaugCgaaaacugcu.................	9	1	Fm3
....uAagaucaugugaaaacugcu.................	4	1	Fm3
....Cgagaucaugugaaaacugcu.................	1	1	Fm3
....ugagaucaugugaaaacuCcu.................	1	1	Fm3
....ugagaucaugugaaaGcugcu.................	1	1	Fm3
....ugagaucaugugaaUacugcu.................	1	1	Fm3
....ugagaucaugugaaaacugcA.................	11	1	Fm3
....ugagaucaugugaaaacugUu.................	5	1	Fm3
....ugagaAcaugugaaaacugcu.................	2	1	Fm3
....ugagaucaugugaaaaGugcu.................	10	1	Fm3
....ugagaucaugugaaaaAugcu.................	10	1	Fm3
....ugagauUaugugaaaacugcu.................	3	1	Fm3
....ugagaucaugugaGaacugcu.................	2	1	Fm3
....ugagaucaugugaaaacugAu.................	1	1	Fm3
....ugagauAaugugaaaacugcu.................	1	1	Fm3
....ugGgaucaugugaaaacugcu.................	4	1	Fm3
....uCagaucaugugaaaacugcu.................	1	1	Fm3
....Agagaucaugugaaaacugcu.................	1	1	Fm3
....ugagaucaugugaaaacugcu.................	5772	0	Fm3
....ugagaucaugGgaaaacugcu.................	6	1	Fm3
....ugagCucaugugaaaacugcu.................	1	1	Fm3
....ugagaucauguUaaaacugcu.................	2	1	Fm3
....ugagaucauUugaaaacugcuu................	1	1	Fm3
....ugagauUaugugaaaacugcuu................	1	1	Fm3
....uAagaucaugugaaaacugcuu................	5	1	Fm3
....uCagaucaugugaaaacugcuu................	2	1	Fm3
....ugagaucaugugaaaacuCcuu................	1	1	Fm3
....ugagaucaugugaaaacGgcuu................	39	1	Fm3
....ugagaucaugugaaaacAgcuu................	44	1	Fm3
....ugagGucaugugaaaacugcuu................	3	1	Fm3
....ugagaucaugugaaaacCgcuu................	50	1	Fm3
....ugagaucaugugaaaaAugcuu................	42	1	Fm3
....ugaCaucaugugaaaacugcuu................	2	1	Fm3
....ugagaucaugugaaaaGugcuu................	13	1	Fm3
....uUagaucaugugaaaacugcuu................	5	1	Fm3
....ugagaucaugugaaaUcugcuu................	1	1	Fm3
....ugagauAaugugaaaacugcuu................	1	1	Fm3
....ugagaucaugugaaaacugUuu................	25	1	Fm3
....ugagaucauAugaaaacugcuu................	2	1	Fm3
....ugagaucaugugaaaacugcCu................	4	1	Fm3
....ugagaucaugugaaaacugcuA................	9	1	Fm3
....Cgagaucaugugaaaacugcuu................	2	1	Fm3
....ugagaucaugugaaaacugcuu................	9949	0	Fm3
....ugUgaucaugugaaaacugcuu................	3	1	Fm3
....ugagaucaugugaaGacugcuu................	2	1	Fm3
....Ggagaucaugugaaaacugcuu................	1	1	Fm3
....ugaAaucaugugaaaacugcuu................	1	1	Fm3
....ugagCucaugugaaaacugcuu................	2	1	Fm3
....ugagaucauCugaaaacugcuu................	1	1	Fm3
....ugagaucaugAgaaaacugcuu................	14	1	Fm3
....ugagaucaugugaaaacugcuC................	2	1	Fm3
....ugagaucauguAaaaacugcuu................	2	1	Fm3
....ugagaucaugugaaaaUugcuu................	19	1	Fm3
....ugagaucaugugaaUacugcuu................	1	1	Fm3
....ugagaucaugCgaaaacugcuu................	9	1	Fm3
....ugagUucaugugaaaacugcuu................	1	1	Fm3
....Ngagaucaugugaaaacugcuu................	3	1	Fm3
....ugagaucauguCaaaacugcuu................	1	1	Fm3
....ugagaucUugugaaaacugcuu................	2	1	Fm3
....ugagaucaugugaaaacuAcuu................	1	1	Fm3
....ugagaucaugugaaaacugGuu................	1	1	Fm3
....ugagaCcaugugaaaacugcuu................	1	1	Fm3
....ugagaucaugugaGaacugcuu................	1	1	Fm3
....ugagaucaugugaaaacuUcuu................	1	1	Fm3
....ugagaucaugugUaaacugcuu................	2	1	Fm3
....ugagaucaugugaaaCcugcuu................	1	1	Fm3
....ugagaucaugGgaaaacugcuu................	8	1	Fm3
....ugGgaucaugugaaaacugcuu................	4	1	Fm3
....ugagaucaCgugaaaacugcuu................	2	1	Fm3

Star **Mature**

ggacauggagcucuaccauaaaagacgaaaccgguuuucauaaugauuuuacagauugauuuugaaacucugagaucaugugaaaacugcuuuugucgacagugauca			
....ugagaucaugugCaaacugcuu................	1	1	Fm3
....ugagaucauguUaaaacugcuu................	5	1	Fm3
....ugagaucaugugGaaacugcuu................	1	1	Fm3
....ugaUaucaugugaaaacugcuu................	1	1	Fm3
....ugagaucaugugaaaaUugcuuu...............	1	1	Fm3
....ugagaucaugAgaaaacugcuuu...............	1	1	Fm3
....Cgagaucaugugaaaacugcuuu...............	1	1	Fm3
....ugagaucauguUaaaacugcuuu...............	1	1	Fm3
....ugagaucaugugaaaaGugcuuu...............	1	1	Fm3
....ugagaucaugugaaaUcugcuuu...............	1	1	Fm3
....ugagaucUugugaaaacugcuuu...............	1	1	Fm3
....ugagaucaugugaaaacCgcuuu...............	1	1	Fm3
....ugagaucaugugaaaacugcuuG...............	3	1	Fm3
....ugagaucaugugaaaacugcuuu...............	915	0	Fm3
....ugagaucaugugaaaacugUuuu...............	7	1	Fm3
....ugGgaucaugugaaaacugcuuu...............	1	1	Fm3
....ugagaucaugugaaaacugcuuA...............	65	1	Fm3
....ugagaucaugugaaaCcugcuuu...............	1	1	Fm3
....ugagaucaugugaaaaAugcuuu...............	2	1	Fm3
....ugagaucaugugaaaacGgcuuu...............	4	1	Fm3
....ugagaucaugugaaaacAgcuuu...............	3	1	Fm3
....ugagaucaugugaaaacugcuAu...............	1	1	Fm3
....ugagaucaugCgaaaacugcuuu...............	1	1	Fm3
....ugagaucaugugaaaacugcGuu...............	1	1	Fm3
....ugagaucaugugaaaacugcuuGu..............	1	1	Fm3
....ugagaucaugugaaaacAgcuuuu..............	1	1	Fm3
....ugagaucaugugaaaacugcuuAu..............	8	1	Fm3
....ugagaucaugugaaaacugcuuuG..............	2	1	Fm3
....ugagaucaugugaaaacugcuuuu..............	100	0	Fm3
....ugagaucaugAgaaaacugcuuuu..............	1	1	Fm3
....ugagaucaugugaaaaUugcuuuu..............	1	1	Fm3
....ugagaucaugugaaaacugcuuuA..............	2	1	Fm3
....ugagaucaugugaaaacugUuuuu..............	1	1	Fm3
....ugagaucaugugaaaacugcuuuuA.............	1	1	Fm3
....ugagaucaugugaaaacugcuuuuU.............	3	1	Fm3
....gagaucaugugaaaacugcu.................	4	0	Fm3
....gagaucaugugaaaacugcuu................	3	0	Fm3
....gagaucauUugaaaacugcuu................	1	1	Fm3
....Uagaucaugugaaaacugcuu................	1	1	Fm3
....gagaucaugugaaaacugcuuu...............	1	0	Fm3
....gagaucaugugaaaacugcuuuu..............	1	0	Fm3
....Ugaucaugugaaaacugcuu................	2	1	Fm3
